# Multi-Criterial Model for Weighting Biological Risk Factors in Multiple Sclerosis: Clinical and Health Insurance Implications

**DOI:** 10.3390/healthcare11172420

**Published:** 2023-08-29

**Authors:** Roberto De Masi, Stefania Orlando, Chiara Leo, Matteo Pasca, Luca Anzilli, Maria Carmela Costa

**Affiliations:** 1 Complex Operative Unit of Neurology, “F. Ferrari” Hospital, Casarano, 73042 Lecce, Italy; 2 Laboratory of Neuroproteomics, Multiple Sclerosis Centre, “F. Ferrari” Hospital, Casarano, 73042 Lecce, Italy; 3 Department of Management, Economics, Mathematics and Statistics, University of Salento, 73100 Lecce, Italy; 4 Complex Operative Unit of Ophthalmology, “V. Fazzi” Hospital, 73100 Lecce, Italy

**Keywords:** Choquet integral, fuzzy algorithm, multiple sclerosis, health insurance, risk factor

## Abstract

The etiology of Multiple Sclerosis (MS) remains undetermined. Its pathogenic risk factors are thought to play a negligible role individually in the development of the disease, instead assuming a pathogenic role when they interact with each other. Unfortunately, the statistical weighting of this pathogenic role in predicting MS risk is currently elusive, preventing clinical and health insurance applications. Here, we aim to develop a population-based multi-criterial model for weighting biological risk factors in MS; also, to calculate the individual MS risk value useful for health insurance application. Accordingly, among 596 MS patients retrospectively assessed at the time of diagnosis, the value of vitamin D < 10 nm/L, BMI (Body Mass Index) < 15 Kg/m^2^ and >30 Kg/m^2^, female sex, degree of family kinship, and the range of age at onset of 20–45 years were considered as biological risk factors for MS. As a result, in a 30-year-old representative patient having a BMI of 15 and second degree of family kinship for MS, the major developmental contributor for disease is the low vitamin D serum level of 10 nm/L, resulting in an MS risk of 0.110 and 0.106 for female and male, respectively. Furthermore, the Choquet integral applied to uncertain variables, such as biological risk factors, evidenced the family kinship as the main contributor, especially if coincident with the others, to the MS risk. This model allows, for the first time, for the risk stratification of getting sick and the application of the health insurance in people at risk for MS.

## 1. Introduction

In spite of the long-standing research efforts, the etiology of Multiple Sclerosis (MS) remains unknown, and its diagnosis is still based on the pathophysiological concept of the dissemination in space and time of brain lesions [1]. In fact, since MS is a multifactorial disease, several pathogenic risk factors have been characterized, but each of these is thought to weight negligibly per se for disease development, assuming, instead, a significant weight when it interacts with all the others. This pathophysiological gap hampers, in the single patient, the clinical risk understanding of MS and its health insurance application. Furthermore, the statistical weight of each risk factor in determining MS when interacting with others is currently elusive. In this context, the aim of this work is to develop a population-based multi-criterial model for weighting risk factors in MS. Moreover, calculating the MS risk (R_MS_) in a single patient is useful to quantify the premium for the health insurance. The selection of considered risk factors takes into account not only their biological relevance, but also their appropriateness as calculation/insurance variables. The latter, in fact, must be equally and adequately distributed in the study population to represent it. However, even considering the great clinical selection of cases, about 25% of the subjects having the pathology may never show it clinically, as demonstrated by coinciding autopsy studies [2]. In all cases, a common condition must be associated with the development of MS and its unfavorable outcome.

Sex is the first and most important studied risk factor, as MS is a female-predominant disease, which expresses a sex ratio of 3:1, increasing over time [3]. In fact, sex affects multiple aspects of MS, including incidence, expression, activity, prognosis, comorbidities, disease outcome and recovery from relapses [4]. Very interesting sex differences emerge when we focus on the male gender in MS. Statistically, men are less likely to develop MS, but if they do, they are more likely to develop the primary progressive MS (PPMS). PPMS is the only MS phenotype that is not predominantly female with a sex ratio of 1:1 [4]. Clinically, men have a worse prognosis disease than women as well as poorer recovery from relapses, with more cognitive and motor impairment, and are less likely to have optic neuritis, resulting in greater disability development and higher transition rates from relapsing-remitting (RRMS) to secondary progressive (SPMS) phenotype [5]. Comorbid conditions in MS are important as they can reduce the central nervous system (CNS) reserve and contribute to accelerating CNS aging [6]. Comorbidities are known to differ based on sex. In fact, diabetes, hypertension, hyperlipidemia, and ischemic heart disease increase with age and are more frequent in males than women affected by MS [7,8].

From the MRI point of view, men have larger brains with a higher percentage of white matter (WM), unlike women who show, in proportion, a higher percentage of gray matter (GM) [9]. However, higher rates of whole brain volume loss and regional GM atrophy (thalamic and cortical), as well as a more advanced impact on cognition were found in men than in women [10]. Consistently, women are affected more frequently by WM atrophy and fewer by cognitive disturbances [11]. Biologically, men with MS are believed to have hypogonadotropic hypogonadism with lower levels of testosterone reported in about 40% of cases [12,13]. This finding is consistent with worse physical and cognitive disability. In summary, men get sick with MS less often, but they get sick more seriously.

Age in MS reflects the risk of the disease development, response to disease-modifying therapy (DMT), disease outcome, and the probability to progression [14]. About 10–15% of cases are diagnosed in the extreme periods of life, <12 and >55 years. In fact, the oldest and pediatric forms are rare, although possible, with the former increasingly on the rise. From the migrant population-based studies, we know that the MS risk follows that of the migrating subject or takes that of the immigration area depending on whether or not the move occurs after or before the 15 years of age, respectively [15]. This evidence suggests an incident age-related environmental factor in the etiology of MS. The age at onset of maximum frequency is 20–45 years [16]. The age of 40–45 years at onset is also important as it is related to opposite clinical characteristics: favorable if less than 40-years-old, associated with females, optic neuritis or sensory symptoms, and relapsing-remitting course with a low attack frequency; unfavorable if onset is over the age of 40, associated with males, motor or cerebellar symptoms, progressive course, and a high number of attacks [17,18]. It is also known that the response to the DMT is less effective after the age of 40. This cut-off takes into account the CNS threshold plasticity in remyelination after inflammatory attacks due to relapse and represents the biological base of the statistical correlation between age, age at onset, disease duration, and neurological disability in MS [18].

Obesity is a sore of modern civilized societies and represents a diffused comorbidity, including MS. It seems to be not only an environmental risk factor for MS, but also an etiological element in the development and evolution of the disease. In particular, childhood and adolescent obesity doubles the risk of MS incidence in both genders, but more so in women [19]. Consistently, Gianfrancesco and coworkers reported in 2017 strong evidence of a causal and independent association between low serum vitamin D levels, increased Body Mass Index (BMI), and MS risk in pediatric age [20]. The biological basis of this association lies in the sharing of the inflammatory component. This also applies to other chronic inflammatory diseases of target organs (cardiovascular system, diabetes, etc.). From a pathophysiological point of view, the sharing of inflammation between MS and obesity is based on proinflammatory molecules secreted by the adipose tissue and the imbalance of gut microbiota. In fact, the adipose tissue is no longer considered to have only a fatty acid/energy storage function, but it is also the place of hypertrophy and subsequent adipocyte hyperplasia characterized by a “chronic low-grade” inflammation, with high levels of proinflammatory mediators [21]. The latter are hormones such as leptin, adiponectin, resistin, or cytokines such as IL6 or TNFα, all having a final molecular way of switching the immune response from the Th-2 anti-inflammatory to the Th-1 proinflammatory one [22]. Obesity is also related to impaired gut microbiota and it leads to decreased bioavailability of vitamin D regardless of age or ethnicity [23,24].

Hypovitaminosis-D and propionic fatty acid are related in turn to a proinflammatory status with neuroinflammation and recurrent relapses [25]. Many studies have highlighted hypovitaminosis-D as a risk factor for many pathologies such as MS, asthma, cardiovascular disease, and psychiatric diseases [26]. However, whether this deficient condition is the cause or the effect of these diseases remains the focus of intense research.

In any case, vitamin D is thought to be an immunomodulatory agent, which confers a protective effect in MS. In particular, high serum concentrations of 25-hydroxycholecalciferol (75 nm/L) are correlated with a reduced risk of MS; on the contrary, low serum levels of vitamin-D with an increased MS risk [27,28]. The pathophysiological basis of these findings is currently elusive, but it is known that the vitamin-D receptor (VDR) is expressed in immune cells of the adaptive and innate immune system.

In MS, kinship is very important. The literature strongly supports the involvement of genetic factors in the etiology of MS. In fact, a family history of MS has been reported in 15–20% of MS patients, a much higher percentage than in the general population. Monozygotic twins show a significantly higher clinical concordance rate (25–30%) than dizygotic twins (3–7%); the lifetime risk of MS in first-degree relatives of MS index cases is estimated at 3% (4% for siblings, 2% for parents, 2% for children), or threefold greater than the risk for second-degree and third-degree relatives (1%), and 10- to 30-fold greater than the risk in the general population (0.1–0.3%) [29,30]. In addition, affected fathers have been reported to be more likely to have MS than those of affected mothers. This has been attributed to the Carter effect, which is common in polygenic disorders but debated in MS. It is clear, however, that the risk of MS increases with the genetic load and, therefore, it is significantly higher if both parents have MS or in first-degree relatives. All these observations are typical of a multifactorial disease, which occurs through susceptibility genes and their segregation, based on the family kinship. The HLA-DRB1*15:01 haplotype of the MCH-II expresses an odd ratio (OR) of about 3; while the HLA-A02 of the MCH-I is protective with an OR of about 0.6 [31]. Both alleles act by allowing for optimal binding in their own HLA pocket, resulting in antigen presentation in the former, and enhancement of CD8 activity due to cross-stimulation of myelin-reactive T-cell receptor in the latter. The imbalance between genes induces susceptibility to getting sick and, in a chronic inflammatory *milieu* due to obesity and/or hypovitaminosis D, can trigger the onset of the disease.

As can be seen, the only quantifiable risk of MS derives from kinship and sex, unlike the others that are evaluable through mathematical approximations from the MS population study observations.

For this purpose, the Fuzzy algorithm represents an excellent solution in the aggregation analysis of multiple variables [32,33]. However, due to the approximate nature of the same, its application is very difficult. The solution to this problem could be the Choquet integral. Choquet was a French mathematician who, in 1953, studied a method for generally describing approximate variables, such as biological ones [34]. Therefore, the application of the Fuzzy algorithm and Choquet integral to the biologically relevant variables in MS constitutes, in fact, a multi-parametric model for the study of risk factors of the disease. This is also the aim of the present work. Specifically, traditional aggregation functions, such as the weighted average, assume that the factors are independent and that the effects of the factors are additive. As also pointed out by Dujmović and coworkers [35], severity-rating scales used in clinical practice are usually based on a simplistic additive scoring. Although these scales enjoy wide acceptance due to their ease of administration, the additive scoring approach yields indicators of insufficient precision [36,37,38]. In order to model the interrelationships among factors, in this study we develop an MS disease risk index based on the Choquet integral operator. These aims seek to address crucial unmet needs related to risk stratification in MS. Although the biological basis of susceptibility to developing MS is widely understood, accurately quantifying the MS risk currently remains a challenge, especially in the single subject. Conversely, these problems are partially resolved in other fields of medicine, such as cancer and cardiovascular diseases.

Furthermore, the proposed risk index of MS occurrence, the RMS index, could be used to obtain an aggregated assessment of MS risk in health insurance systems whose premiums are calculated on the basis of a few individuals’ medical information regarding vital signs, health status, and lifestyle indicators. For example, insurance companies might design prepaid health insurance plans that offer differentiated premiums based on the associated risk diagnosis. However, an accurate actuarial model requires a wide range of statistical data which, in practice, are only available in the aggregate form that is proposed here.

A direct outcome of the risk stratification study in MS is the health insurance. Essentially, an effective risk stratification model improves our ability to predict pathological events and subsequent neurological impairments over a person’s lifetime. This study also considers this practical implication of clinical findings reached by the proposed model.

## 2. Materials and Methods

### 2.1. Study Design

In an open-label, cross sectional, retrospective and real-world study, we enrolled 700 consecutive MS patients who were not selected by age, sex, or ethnicity, and who were afferent to the Multiple Sclerosis Centre of Neurological Department at the “F. Ferrari” Hospital in Casarano, Lecce (Italy). Each enrolled subject subscribed written informed consent and was investigated for BMI, serum vitamin D levels, age at onset, and self-referred MS family kinship expressed at the time of diagnosis. The family kinship ranging from the first to second degree was considered more penetrating or less penetrating from the third degree onwards.

### 2.2. Study Population

Based on adequate information, the subjects’ enrollment took place at the MS Centre of Casarano (Lecce, Italy) during routine visits, with reference only to inclusion/exclusion criteria.

Inclusion criteria: diagnosis of relapsing remitting or secondary progressive MS according to the 2017 McDonald criteria [1], clear indications on the considered variables expressed at the time of diagnosis. Specifically, we also included patients who, at the time of diagnosis, exhibited a build-up of disabilities due to a delay in diagnosis or treatment. This is because RRMS and SPMS phenotypes share a common biological basis, as they evolve into distinct disease stages. Conversely, we did not consider the amount of the disability or its development, only the occurrence of the disease event being relevant in the study.

Exclusion criteria: diagnosis of primary progressive MS according to the 2017 McDonald criteria [1]. In fact, PPMS patients are thought to be biologically different from other disease phenotypes. Furthermore, any comorbidities (cardiovascular, immunological and metabolic, such as atheromasia or prior stroke, diabetes, arteritis, rheumatoid arthritis, and connective tissue disorders), as well as local or systemic transient inflammatory and septic conditions (bacterial infections, cold, cough, flu, exanthematous diseases and viroses, relevant trauma, smoking) acting at the time of diagnosis. Finally, given the innovative character of the study, we have not considered, for the moment, the EBV serological status and the smoking habit. In fact, EBV antibodies are present in almost 100% of people with adult MS and their titration varies depending on the provenience laboratory; moreover, smoking is a variable distributed in a heterogeneous way between sex and age. Therefore, we referred to the classical risk factors to reduce putative confounding factors.

### 2.3. Statistical Analysis

For the descriptive statistic, the SPSS22 software was used and the non-parametric Wilcoxon-Mann-Whitney test was applied to the difference between means. The confidence interval (CI) of 95% was also calculated for quantitative variables. The inferential statistic was performed with the “R” software with the following theoretic assumptions.

### 2.4. Fuzzy Membership Functions

Specifically, we address mathematically the problem of inferring the degree of MS disease risk associated with a patient’s medical condition considering as baseline factors four main individual risk factors, namely Age at onset, Vitamin D serum levels, BMI, and MS family kinship. Sex was also considered, as we applied the method separately for females and males. For convenience of notation, we denote by N = {1, 2, 3, 4} the index set of factors, being *i* = 1 the Age at onset, *i* = 2 the Vitamin D serum levels, *i* = 3 the BMI, and *i* = 4 the MS family kinship. In this way, a patient’s medical condition can be described by the vector (x_1_, x_2_, x_3_, x_4_), where xi denotes the value of the factor *i*. The methodology that we propose consists of two steps:

(a) determination of individual factors’ risk evaluations r_1_, r_2_, r_3_, r_4_ by using intra-criterion information;

(b) elicitation of the non-additive measure *m* and construction of the overall risk index R_MS_ by using inter-criteria information.

At step (a), we first elicit, for each factor *i* = 1, 2, 3, 4, the relative membership function µ*_i_*(x) representing the risk degree when the value of factor *i* is equal to x. Then, r*_i_* = µ*_i_*(x*_i_*) is the risk degree associated with the patient’s factor value xi. At step (b), we define the MS overall risk index R_MS_ by combining the information expressed by the individual factors’ evaluations r_1_, r_2_, r_3_, r_4_ and the non-additive measure *m* that reflects the weights of the considered risk factors and their interaction. We adopt the Choquet integral as an aggregation operator due to its ability to consider the importance of both individual factors and the interrelationships between them. Consequently, we define the MS overall risk index R_MS_ (x_1_, x_2_, x_3_, x_4_) = C*_m_* (r_1_, r_2_, r_3_, r_4_), where C*_m_* (r_1_, r_2_, r_3_, r_4_) is the Choquet integral of the vector (r_1_, r_2_, r_3_, r_4_) computed with respect to the non-additive measure *m*.

In the following, we explain the proposed methodology in detail.

The severity range of the considered factors can be described by four fuzzy sets represented by

- Age at onset → 20–45 years;
- Vitamin D serum levels → <10 nm/L;
- BMI → <15 Kg/m^2^ and >30 Kg/m^2^ (underweight and obesity, respectively);
- MS family kinship → =1.

As regard to kinship, in this study, it was considered up to the sixth grade, i.e., from parents to children of the parents’ cousins.

Mathematically, a fuzzy set E is described by a membership function μ_E_: X → [0, 1], where μ_E_(x) is the degree of pertaining of x (the distribution of the risk factor in the study population) to the set E (the set of subjects considered at risk). Thus, we associate each individual risk factor *i* (with *i* = 1, 2, 3, 4) with a membership function μ*_i_*: X*_i_* → [0, 1], that describes the risk degree μ*_i_*(x) when the value of factor *i* is equal to x, where μ*_i_*(x) = 1 if x is an extremely altered value and μ*_i_*(x) = 0 if x belongs to its normal range.

In the case of age at onset, the respective membership function μ_1_(x), denoted by *A*(*x*), is given by:

(1) $$A\left(x\right)=\left\{\begin{array}{l} \;0\;if\;x=0\\ \frac{x}{20}\;if\;0<x<20\\ 1\;if\;20\leq x<45\\ 1-\frac{x-45}{25}\;if\;45\leq x<70\\ 0\;if\;x\geq 70 \end{array}\right.$$

In the case of vitamin *D* serum levels, *D*(*x*):

(2) $$D\left(x\right)=\left\{\begin{array}{c} 1\;if\;0\;\leq x<10\\ 1-\frac{x-10}{20}\;if\;10\leq x<30\\ 0\;if\;x\geq 30 \end{array}\right.$$

In the case of BMI, *M*(*x*):

(3) $$M\left(x\right)=\left\{\begin{array}{c} \;0.3\;if\;0\;\leq x<15\\ 0.3+0.7\cdot \frac{x-15}{15}\;if\;15\leq x<30\\ 1\;if\;x\geq 30 \end{array}\right.$$

In the case of family kinship, *K*(*x*):

(4) $$K\left(x\right)=\left\{\;\begin{array}{c} 1\;if\;0\;\leq x<1\\ 1-\frac{x-1}{5}\;if\;1\leq x<6\\ 0\;if\;x\geq 6 \end{array}\right.$$

**Figure 1:**
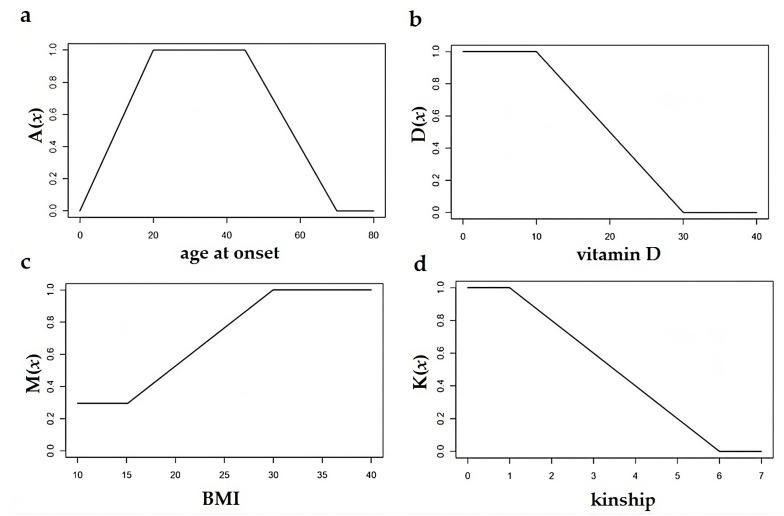
Fuzzy membership functions of age at onset (**a**), vitamin D serum levels (**b**), BMI (**c**), and family kinship (**d**).

In Figure 1, these four Fuzzy membership functions are represented.

We denote by r*_i_* = μ*_i_* (x*_i_*) the risk degree corresponding to the factor value x*_i_*, i.e., r_1_ = A(x_1_), r_2_ = D(x_2_), r_3_ = M(x_3_), and r_4_ = K(x_4_). In this way, we associate the vector of assessments of individual risk factors (r_1_, r_2_, r_3_, r_4_), where r*_i_* is between 0 and 1, with respect to each patient’s medical condition (x_1_, x_2_, x_3_, x_4_).

We now deal with the problem of eliciting the non-additive measure *m* that estimates the weights of the considered risk factors and their interaction.

We consider the index set of factors N = {1, 2, 3, 4} and the set of the parts of N defined by T = {Ø, {1}, {2}, {3} {4}, {1, 2}, {1, 3}, {1, 4}, {2, 3}, {2, 4}, {3, 4}{1, 2, 3}, {1, 2, 4}, {1, 3, 4}, {2, 3, 4}, {1, 2, 3, 4}}, i.e., T is the family of all subsets of N. Observe that T contains 2^4^ elements.

A non-additive measure *m*: T → [0, 1] on the index set N is a monotone measure satisfying the following properties:

- *m*(Ø) = 0
- for all S1, S2 subsets of N, if S1 ⊆ S2 then *m*(S1) ≤ *m*(S2).

We specify that a subset S ⊆ N refers to a medical condition in which all factors *i* ∈ S highlight extremely abnormal values and all other factors *i* ∈ N\S are inside the normal range. In this formal setting, the amount *m*(S) represents the disease risk degree corresponding to such a medical condition.

For instance, the subset S = {1, 3} refers to a medical condition in which factors 1 (age at onset) and 3 (BMI) are far outside their normal range and values of factors 2 (vitamin D serum levels) and 4 (MS family kinship) are normal. Accordingly, *m*(S) = *m*({1, 3}) indicates the MS risk degree corresponding to such a medical condition.

### 2.5. “m” as Estimating Risk Factor Variable in MS

The non-additive measure *m* was obtained by the descriptive statistic, so that by the observation of the distribution of each variable in the study population. Indeed, *m*({1}), *m*({2}), *m*({3}), *m*({4}) is the proportion of the risk factor in the population study; *m*({1, 2}), *m*({1, 3}), *m*({1, 4}), *m*({2, 3}), *m*({2, 4}), *m*({3, 4}) is the proportion of the pairs of the risk factors; *m*({1, 2, 3}), *m*({1, 2, 4}), *m*({1, 3, 4}), *m*({2, 3, 4}) is the proportion of the triplets of the risk factors, and *m*({1, 2, 3, 4}) is the proportion of the risk factors considered all together. In Table 1, we report the non-additive measure m on the index set N for females and males.

### 2.6. The Choquet Integral Applied to MS

Now, we can associate an overall MS risk evaluation to a patient’s medical condition (x_1_, x_2_, x_3_, x_4_) by aggregating the individual factors’ risk evaluations r_1_, r_2_, r_3_, r_4_ with the information provided by the non-additive measure *m* on the influence of each single risk factor and on the interactions between factors. We perform the aggregation process by using as aggregation operator the Choquet integral, since it is an appropriate substitute to the weighted arithmetic mean to aggregate dependent risk factors.

We define the overall MS risk index R_MS_ as: R_MS_ (x_1_, x_2_, x_3_, x_4_) = *C_m_* (r_1_, r_2_, r_3_, r_4_) where *C_m_* (r_1_, r_2_, r_3_, r_4_) is the Choquet integral of the vector (r_1_, r_2_, r_3_, r_4_) computed with respect to the non-additive measure *m*.

It can be calculated using the formula

*C_m_* (r_1_, r_2_, r_3_, r_4_) = r_σ(1)_ [*m*({σ(1), σ(2), σ(3), σ(4)}) − *m*(σ(2), σ(3), σ(4))]
+ r_σ(2)_ [*m*(σ(2), σ(3), σ(4)) − *m*(σ(3), σ(4))]            
+ r_σ(3)_ [*m*(σ(3), σ(4)) − *m*(σ(4))]                 
+ r_σ(4)_ *m*(σ(4))                         (5)

where σ: {1, 2, 3, 4} → {1, 2, 3, 4} is a suitable permutation of indices {1, 2, 3, 4} such that r_σ(1)_ ≤ r_σ(2)_ ≤ r_σ(3)_ ≤ r_σ(4)_.

### 2.7. The R_MS_ Application in Health Insurance

The calculation of the R_MS_ index was carried out by implementing a code using the R software. It provides the risk of subject to develop MS based on each patient’s medical condition.

The R_MS_ index will be used to provide the amount of the insurance premium according to the following formula: P_MS_ = *p_min_* + *β*·(*p_max_* − *p_min_*), where *p_max_* and *p_min_* are the maximum and minimum premium required by the insurance company. The maximum premium represents the sum to be paid in the event that the subject gets the disease. *β* represents the MS risk for a single subject and it is expressed by *β* = α + R_MS_ (1 − α) where α is the proportion of population already affected by MS and R_MS_ is the likelihood of getting sick. In this work, we will calculate the P_MS_ according to an R_MS_ of a typical subject (male and female), age 30, who has no comorbidities and expresses a medium BMI of 15 Kg/m^2^ and a serum vitamin D level of 20 nm/L, at the lower CI limits, and a family kinship of 2.

## 3. Results

Among the study population of 229 M and 471 F patients affected by MS, 56 patients (25 M, 31 F) were excluded as affected by PPMS, 14 (eight M, six F) by comorbidities, and 34 (20 M, 14 F) because they were smokers. In the remaining 596 patients, we observed a sex ratio F:M of 2.1:1.0 with mean age at onset of 32 years (min 11, max 65, SD 11.8, CI 95% 20–45 years). Furthermore, contextual mean serum vitamin D level was 28 nm/L (min 10, max 52, SD 8.9, CI 95% 10–30 nm/L); mean BMI was 17 Kg/m^2^ (min 14.3, max 36.7, SD 8.6, CI 95% 15–30). Sixty-five patients expressed an MS family kinship, of which 12 in the first degree and 26 in the second. The remaining patients had the following family kinship: eight patients the third, eight the fourth, six the fifth, and five the sixth degree. Table 2 summarizes these data.

**Figure 2:**
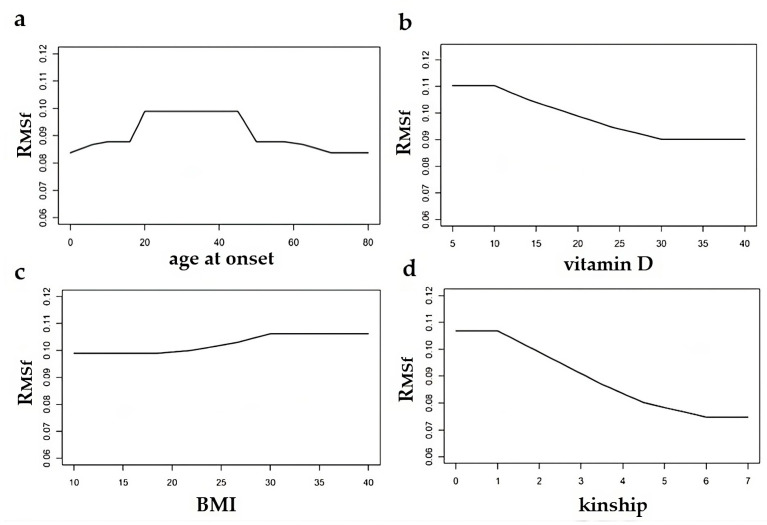
Variation of the output of each risk factor individually, assuming that the others do not change. Specifically, a change of the MS risk in females (R_MSf_) with age at onset (**a**), vitamin D serum levels (**b**), BMI (**c**), and family kinship (**d**), with the other three factors fixed.

**Figure 3:**
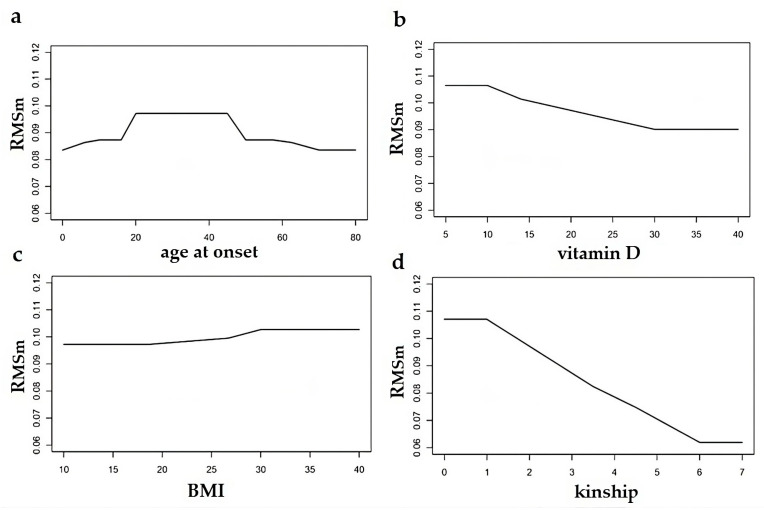
Variation of the output of each risk factor individually, assuming that the others do not change. Specifically, a change of the MS risk in males (R_MSm_) with age at onset (**a**), vitamin D serum levels (**b**), BMI (**c**), and family kinship (**d**), with the other three factors fixed.

The multi-parametric model returns the corresponding statistical weight for female and male gender (Figure 2 and Figure 3).

In Figure 2 and Figure 3, we show the R_MS_ index referring to females (Figure 2) and males (Figure 3) as a function of an individual risk factor, by fixing the other ones. Specifically, in Figure 2a and Figure 3a, the R_MS_ index is plotted as a function of the age at onset, with x_2_ = 20 (vitamin D serum level), x_3_ = 15 (BMI), x_4_ = 2 (family kinship). In Figure 2b and Figure 3b, the R_MS_ index is plotted as a function of the vitamin D serum level, with x_1_ = 30 (age at onset), x_3_ = 15 (BMI), x_4_ = 2 (family kinship). In Figure 2c and Figure 3c, the R_MS_ index is plotted as a function of the BMI, with x_1_ = 30 (age at onset), x_2_ = 20 (vitamin D serum level), x_4_ = 2 (family kinship). In Figure 2d and Figure 3d, the R_MS_ index is plotted as a function of the family kinship, with x_1_ = 30 (age at onset), x_2_ = 20 (vitamin D serum level), x_3_ = 15 (BMI).

Note the detectable slope increase in both R_MSf_ and R_MSm_ curves as the enhancing effect of the 20–45 age range on the risk of getting MS (Figure 2a vs. Figure 3a), although there is a slight difference in men as compared to women. On the contrary, in the case of vitamin D, the slope is quite constant due to the lack of influence on the R_MSf_ and R_MSm_ values of the serum level of the metabolite. The BMI also shows a quite constant slope curve, but it increases starting from the value of 30 Kg/m^2^ with a negligible intergender difference. This kind of threshold effect of risk factors is also obtained considering the degree of kinship, but unlike the other risk factors, the variation in this function is more noticeable. In fact, the R_MSm_ decreases more rapidly starting from the value associated to the first degree of kinship; on the contrary, the R_MSf_ curve tends to stabilize after the first kinship, and its slope is less than the male one (Figure 2d vs. Figure 3d).

**Figure 4:**
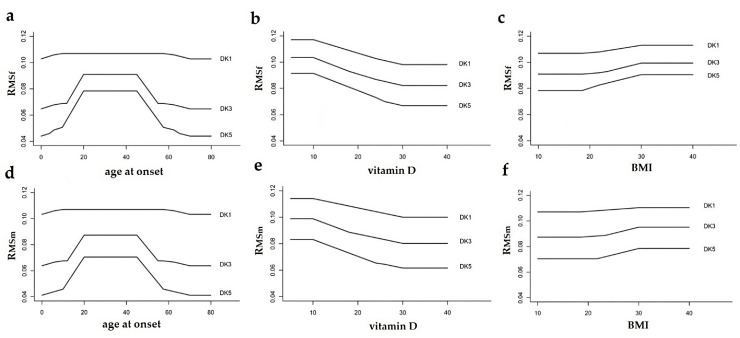
Variation of age at onset (**a**,**d**), vitamin D serum levels (**b**,**e**), and BMI (**c**,**f**) as the degree of kinship varies from first, third, and fifth (DK1, DK3, and DK5) with the other two variables fixed, and the resulting effect on MS risk for the female (R_MSf_, **top**) and male (R_MSm_, **bottom**) genders.

At this point, we have also determined the R_MSf_ and R_MSm_ as outputs since the kinship varies from the first to fifth degree. Specifically, how the age at onset, vitamin D serum level, and BMI change with the degree of kinship and its effect on the R_MS_ (Figure 4).

Note the detectable effect of the 20–45 age range on the kinship 3 to 5, resulting in R_MSf_ and R_MSm_ value elevation. This effect of age becomes negligible on the kinship 1, due to its preponderance in determining MS risk. The intergender difference was not significant. In the case of vitamin D, the slope is lowered and quite constant, starting from the value of 20 nm/L or more. In fact, both R_MSm_ and R_MSf_ assume maximum levels when vitamin D levels are 20 nm/L or less, due to their pathogenic weight. The latter also express a negligible intergender difference.

This finding applies if kinship ranges from the third to fifth degrees, but not the first. The role of kinship 1 is predominant over that of vitamin D, resulting in a constant slope that is independent of plasma levels of the latter. BMI also evidences a quite constant slope curve, but it increases starting from the value of 30 with a detectable difference in females expressing more concordant values. However, BMI is affected by the kinship influence, always equal from the first to fifth degrees of family.

In Table 3 we compute the R_MSf_ and R_MSm_ for different female and male patients’ risk factors, such as decreasing vitamin D serum level in conjunction with age at onset (p1–p4), BMI (p5–p8), and family kinship (p9–p12), having, each time, the other two factors fixed. For example, note the elevation of the minimum risk value in conjunction with abnormal values of the coincident risk factors such as vitamin D serum level < 20 nm/L, BMI > 30 Kg/m^2^, and family kinship coming from degree 6 to 1. Obviously, age at onset cannot be thought to assume normal or abnormal values, but a risk ranging from 20 to 40 years. Consistently, we found the minimum risk value in a patient having normal values of BMI and vitamin D serum level with age at onset other than that of the risk range as well as the family kinship distant from degree 1.

**Figure 5:**
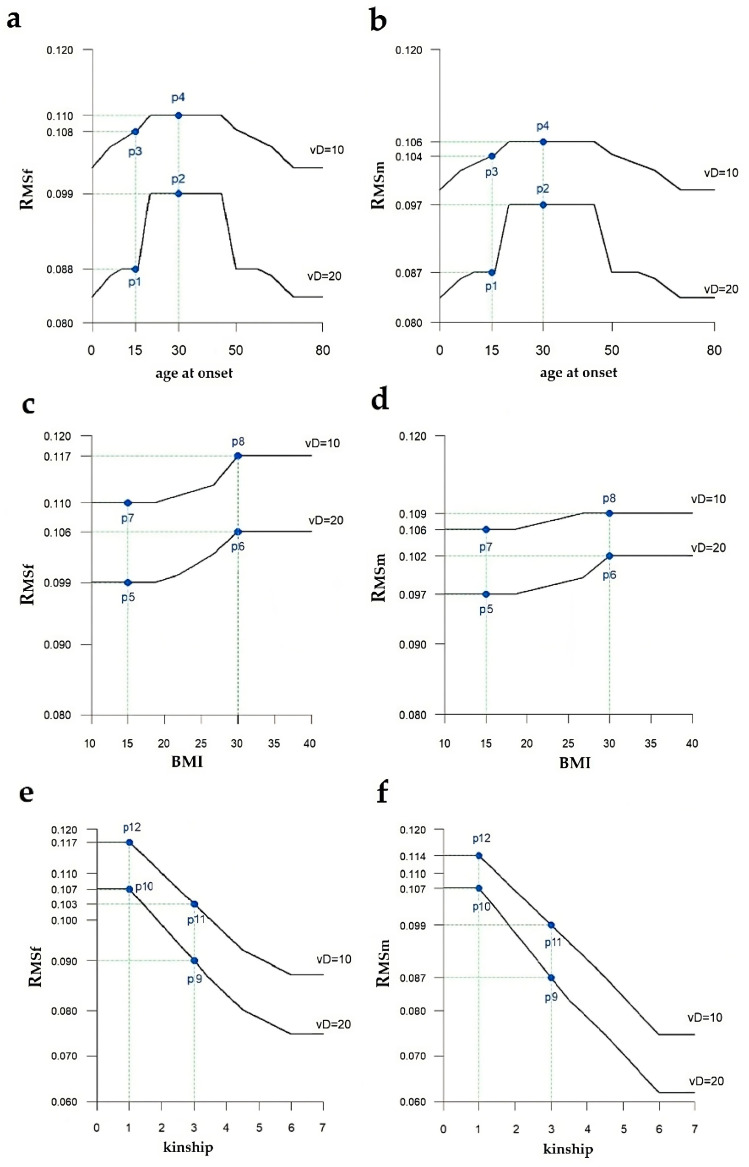
R_MS_ index referred to Age at onset (**a**,**b**), BMI (**c**,**d**), and kinship (**e**,**f**) by fixing two vitamin D serum levels (vD) from 20 to 10 nm/L, assuming that the others do not change. Specifically: (**a**,**b**) R_MS_ index referred to age at onset in females (**a**, R_MSf_) and males (**b**, R_MSm_) with p1–p4 patient cases expressing a change of the MS risk in patients having age at onset of ages 15 (p1, p3) and 30 (p2, p4) and decreasing vitamin D serum levels (vD) from 20 to 10 nm/L, with fixed BMI of 15 Kg/m^2^; in addition, degree 2 of family kinship. (**c**,**d**) R_MS_ index referred to BMI in females (**c**, R_MSf_) and males (**d**, R_MSm_) with p5–p8 patient cases expressing a change of the MS risk in disease subjects having BMI of 15 Kg/m^2^ (p5, p7) and 30 Kg/m^2^ (p6, p8) with decreasing vitamin D serum levels (vD) from 20 to 10 nm/L. (**e**,**f**) R_MS_ index referred to kinship in females (**e**, R_MSf_) and males (**f**, R_MSm_) with p9–p10 patient cases expressing a change of the MS risk in patients having degree 3 of family kinship (p9, p11) and degree 1 (p10, p12) with decreasing vitamin D serum levels (vD) from 20 to 10 nm/L.

The variations of the output R_MSf_ and R_MSm_ are graphically represented in Figure 5, where p1 represents the patient case at the minimum risk and p2–p12 represents the other patients cases at increased R_MS_, resulting from the coincident risk factors (see caption for detailed combinations).

In Figure 5, we show the R_MS_ index referred to females (a,c,e) and males (b,d,f) as a function of an individual risk factor, by fixing the other ones. Specifically, in Figure 5a,b, R_MSf_ and R_MSm_ indexes are plotted as a function of the x_1_ ranging from 15 to 40 years (Age at onset), x_2_ assuming values 20 or 10 nM/L (vitamin D serum level), x_3_ = 15 (BMI) and x_4_ = 2 (degree 2 of family kinship). Note the detectable slope increase in both R_MSf_ and R_MSm_ curves as an enhancing effect of the 15–40 age range on the risk of getting the disease, although there is a slight difference in men as compared to women. Furthermore, in the case of vitamin D, the curve slope values are different between 10 and 20 nm/L, due to the detectable influence on the R_MSf_ and R_MSm_ of the serum metabolite levels, with a slight intersex difference.

The BMI also shows a quite constant curve slope, but it increases, starting from the value of 30 Kg/m^2^ with a negligible gender difference (Figure 5c,d).

This kind of threshold effect of risk factor is also obtained considering the degree of kinship, but unlike the other risk factors, the variation in this function is more noticeable. In fact, the R_MSm_ decreases more rapidly, starting from the value associated to degree 1 of family kinship; on the contrary, the R_MSf_ curve stabilizes on the left of graph, after degree 1 of family kinship, and its slope is less steep than the male one (Figure 5e,f).

**Figure 6:**
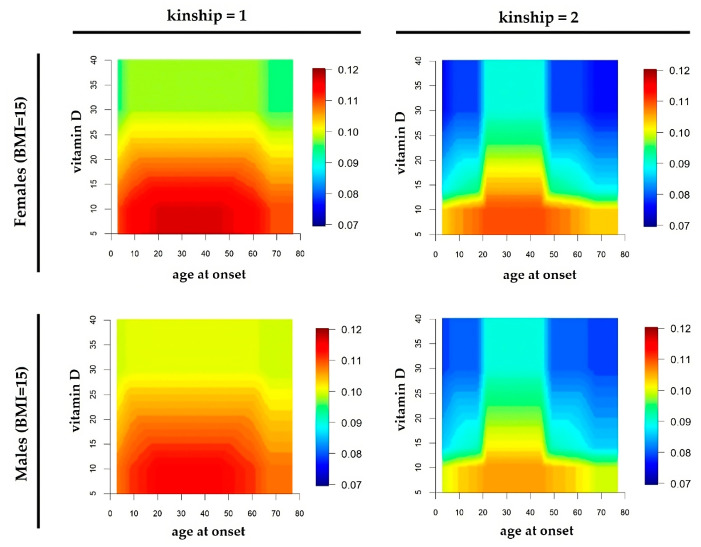
Correlation between considered risk factors such as age at onset and vitamin D serum level with a constant BMI of 15. Color code bars return, finally, the R_MSf_ and R_MSm_ values for degree 1 and 2 of family kinship.

Figure 6 describes graphically the correlation between considered risk factors, returning, finally, the R_MSf_ and R_MSm_ values for degree 1 and 2 of family kinship. Note that the highest R_MSf_ and R_MSm_ values corresponding to vitamin D serum level <20 nm/L and age range 20–40 years, having BMI 15 and degree 1 of family kinship.

Finally, on the basis of R_MSf_ and R_MSm_, we calculated the insurance premium according to the P_MS_ = *p_min_* + *β*·(*p_max_* − *p_min_*) and *β* = α + R_MS_ (1 − α), where α is the MS population as quantified by the Italian National Statistical Institute (ISTAT) in 122.000 subjects at the latest determination, taking into account the MS risk.

Thus, α = 122,000/60,359,546 × 10^6^ = 0.002, 1 − α = 0.998 and given *β_f_* = 0.002 + 0.998 × 0.100818 = 0.1026 for the female gender and *β_m_* = 0.002 + 0.998 × 0.09529266 = 0.0971 for the male one, we have obtained the P_MS*f*_ = *p_min_* + 0.1026 × (*p_max_* − *p_min_*) and P_MS*m*_ = *p_min_* + 0.0971 × (*p_max_* − *p_min_*) that are the MS insurance premiums for females and males, respectively.

## 4. Discussion

Despite the worldwide research effort, MS still remains the main non-traumatic cause of juvenile disability with unknown etiology. Due to this condition, the diagnosis is based on the physiopathological concept of dissemination in time and space of the CNS lesions. For this purpose, a number of epidemiological and biological studies have been made to individuate the MS risk factors. Epidemiological and NGS studies have found that age, family kinship, and some genetic markers, such as HLA 1501, are elements of susceptibility to getting sick [16,30]. Biological observations have indicated some interesting molecules, including vitamin D [26,39], Neurofilament light (NfL) [40,41], IFP35, and GANAB [42,43,44,45] as emerging biomarkers in demyelinating diseases. Each of these variables has been deeply studied in MS literature, but their resulting effects on getting sick when they are coincident is still lacking and unquantified. To this end, we applied the Choquet integral as a core of our statistical model. The Choquet integral finds its main application in decision theory and in imprecise probability theory. Here, it works as a tool of measuring the expected utility of an uncertain event through the study of its membership functions. For this reason, we refer to the latter, considering risk factors as biological variables with an uncertain characteristic.

On these bases, the present work is a real-world, population-based study that retrospectively identified the pathological weight attributed to some biological variables acting as MS risk factors. These variables were assessed in both manners: alone, with the others being fixed, as well as coincident with each other. Specifically, these variables were drawn out of the literature, as they are believed to be independently associated with MS, enough to predict it. However, the extent of this association has not been studied enough, either for the single variable or for the interaction between several of them. In fact, when a variable expresses a characteristic of association with respect to a given disease in order to predict it, this variable can be considered a risk factor. More risk factors acting with each other can induce the development of a disease with a higher likelihood than a single risk factor acting alone. Understanding this likelihood represents a clinically relevant milestone that can be useful in the insurance sciences. This also applies to MS. This epidemiological approach already exists for many diseases, including the ischemic cardiopathy, stroke, and cancer, but is lacking for MS and other autoimmune organ-specific clinical conditions.

As regards MS, the risk of getting sick deriving from the family kinship is the only known one, unlike the others, which are still elusive, especially in interplaying conditions. From this point of view, the idea of using the Fuzzy algorithm in order to aggregate biological variables and consider them with their approximate values through the Choquet integral is innovative and real-world pertaining. In fact, the reference values of the membership variables deduced from our study population are not theoretical, but population-based. Therefore, the study population expresses the characteristics of the *pure* RRMS patient, not affected by comorbidities, nor by the PP form, who were then assessed for BMI, family kinship, age at onset, and vitamin D serum levels. These variables were evaluated both alone and interactively in order to best describe the MS risk.

Considered alone as well as in an interacting model, the MS risk factors express low intergender variability. Specifically, R_MSf_ and R_MSm_ assume the values of 0.099 and 0.097, respectively, corresponding to an age range of 20–40 years and normal vitamin D level of 20 nm/L. These values decrease quickly in the extreme ages of life, reaching the lowest values of 0.088 and 0.087, respectively.

As regards vitamin D, if the metabolite assumes an abnormal level of 10 nm/L, R_MSf_ and R_MSm_ increase gradually with the age range 20–40 years, up to 0.110 and 0.106, respectively. BMI is associated with a gradual increase of R_MSf_ and R_MSm_ going from 15–20 to 30 Kg/m^2^, leading to values of 0.106 and 0.102, respectively, with a normal vitamin D level of 20 nm/L, and values of 0.117 and 0.109, with an abnormal vitamin D level of 10 nm/L. It is interesting to note that R_MS_ conferred by kinship is not affected by gender, age, or other variables. However, this applies only for degree 1 of family kinship. In fact, the family kinship is associated with the values of 0.107 of R_MS_ at degree 1, without intergender difference. Going from degree 1 to degree 5, R_MSf_ and R_MSm_ values decrease quickly to the minimal values, assuming 0.090 and 0.087 at intermediate degree 3, respectively. On the contrary, R_MS_ due to kinship is noticeably affected by vitamin D serum level, up to 0.117 and 0.114 for R_MSf_ and R_MSm_, respectively, and less by BMI. Finally, it is not affected by age at onset, except for in the case of degree 1, as shown in Figure 5.

Considered interactively with each other, the MS risk factor values described in our multi-parametric model are partially affected by BMI, vitamin D serum levels, and age at onset, but mainly by family kinship. The latter alone confers an MS risk which increases if coincident with the others.

The minimum risk value is found for the age at onset of 15 associated with a normal BMI of 15–20 Kg/m^2^ and vitamin D serum level of 20 nm/L. This is the patient case “p1”. Every modification of these parameters affects R_MS_. In fact, R_MSf_ undergoes an increase of 13%, and R_MSm_ increases 11% for the age going from 15 to the risk range of 20 to 40, associated with a normal vitamin D serum level of 20 nm/L and assuming a fixed BMI of 15–20 Kg/m^2^ and degree 2 of family kinship. This is the patient case “p2”.

The major MS risk is conferred by the combination of increasing age with decreasing vitamin D serum level. In fact, R_MSf_ undergoes an increase of 23% and 25%, and R_MSm_ increases 20% and 22% for vitamin D serum levels going from 20 to 10 nm/L and for the association of these two conditions (cases “p3” and “p4”, respectively). Coherently, R_MSf_ of 0.110 of “p4”, age 30, and 10 nm/L of vitamin D serum level is higher than R_MSf_ of 0.099 regarding age alone as a single MS risk factor. The difference of 0.011 between two cases reflects the interaction of vitamin D serum level on the age at onset, resulting in a final enhancement of the MS risk in female subjects.

Furthermore, at the same age of 30 and degree 2 of family kinship, R_MSf_ undergoes an increase of 21% and 33%, and R_MSm_ increases 17% and 25% for an increase in BMI to 30 Kg/m^2^ and when the latter is associated with an abnormal vitamin D serum level of 10 nm/L (cases “p6” and “p8”, respectively).

On the contrary, the family kinship seems to nullify the effect of all other variables, acting as an independent risk factor (cases “p10” and “p12”). This is mainly true for the male gender, since in females, the difference between degrees is very limited. In fact, the R_MSm_ decreases more rapidly, starting from the value associated to degree 1 of family kinship (Figure 5f).

The pathogenic weight of the family kinship quickly decreases in both genders, going from the second degree to the third one, without ever disappearing. This profile is specific for the family kinship and is unusual for the other variables. In particular, the vitamin D serum level expresses the lowest weight, having a substantially flat curve. BMI appears to be relevant after the value of 30 Kg/m^2^. The family kinship also expresses a gradual risk reduction after the first degree. Finally, the age curve highlights a critical risk, ranging from 20 to 45 years old. The risk map in Figure 6 clearly summarizes the correlation between age and a vitamin D serum level, attributing higher R_MS_ in the case of females and first degree of family kinship.

On this basis, it is clear that the maximum risk is attributed to a female subject with a BMI value of 30 Kg/m^2^, aged between 20 and 45 years at MS onset and a first-degree MS family kinship with a <20 nm/L of vitamin D serum level. This risk gradually decreases and stratifies as BMI values normalize, the vitamin D serum level increases, the age moves out of critical range and, above all, the degree of MS family kinship increases after the second one. These findings are useful in the personalized attribution of the MS risk and, therefore, in its stratification. The latter are not a statistically determined theoretical entity, but a biological condition, based on the gene-environment interaction.

Gene-environment interaction occurs when genetic factors affect sensitivity to environmental risk factors, making some people more susceptible [46]. In our model, vitamin D serum level and obesity modulate the susceptibility to get sick, already determined by sex and family kinship. The molecular basis of this modulation is under investigation, but widely attributed to neuroinflammatory enhancement. In fact, obesity, especially in adolescence, is associated with the inflammatory *milieu* and hypovitaminosis D, resulting in a proinflammatory dysregulation. Both these inflammatory conditions, in turn, increase antigen presentation and tissue damage. However, gene-environment interaction is not a physical law, but a biological variable statistically distributed over a large population. This distribution expresses a risk function with a probability of getting sick. This means that two coincident risk factors have a stronger deterministic role than one alone. This explains why some individuals with both environmental and genetic risk factors are not necessarily suffering from MS.

Thus, risk stratification plays a fundamental role in actuarial statistics applied to health insurance. Consistently, the main direct effect of our study is the definition of the insurance premium, according to R_MSf_ and R_MSm_ values. Although this represents an enhancement of our knowledge, this formula P_MS_ = *p_min_* + *β ×* (*p_max_* − *p_min_*) suffers in the *β* element by quality of the study population that could express the general characteristics of the MS population. In fact, the main limitation of this study, in spite of the adequate sample size and the unbiased enrollment, is providing theoretical guarantees of representativeness. In other words, limitations of the proposed method are the membership values’ attribution. These values are population-based and suffer from the biological and demographic characteristics of the latter. For this reason, the proposed method needs a standardization procedure before being applied to the general population. Furthermore, using data from a selected sample of MS patients for retrospective analysis is prone to recall bias in this study. However, a longitudinal study should prospectively observe a number of normal subjects getting sick with a frequency about of 0.04: 1 × 10^3^/year [47], over a few decades. The retrospective approach has also been used in the risk study of cardiovascular diseases. The latter was also observed prospectively in the literature, but the frequency of getting sick about of 17.6:1 × 10^3^/year [48,49] makes their longitudinal observation realistic. This is the explanation for why we adopted a retrospective rather than prospective approach to the study. Actually, it can be asserted that there are currently no specific insurance exigences in MS and demyelinating disease, but it is also true that no insurance offers have emerged in this area so far. Nevertheless, we believe that in the near future there will likely be a demand for likelihood estimates of developing a lifelong neurological disability. This demand is already present in the cardiovascular or oncological fields.

In any case, the present study improves our knowledge in the field of insurance sciences applied to MS. In fact, only by estimating the overall risk can an insurer develop a monthly premium to provide the payment for the health care benefits specified in the insurance agreement in the event that disease occurs. This is the principle upon which health care in the US and Commonwealth countries is based. There is currently a disease-specific health insurance plan for heart attack, cancer, and diabetes [50,51], but not for MS, due to the lack of studies on the issue of predictive values. The latter are important prerequisites, capable of identifying, in a valid, simple, and inexpensive way, the population at risk of MS, which needs proactive and planned care in the event the disease occurs.

## 5. Conclusions

In summary, our multi-parametric model provides a population-based method for the quantitative assessment of the MS risk in the single patient. This MS risk can now be quantified and stratified, as well as used in actuarial statistics to determine the health insurance premiums. Specifically, the maximum MS risk is attributed to a female subject with a BMI value of 30 Kg/m^2^, aged between 20 and 45 years at MS onset and a first-degree MS family kinship, with a <20 nm/L of vitamin D serum level. This risk gradually decreases and stratifies as BMI values normalize, the vitamin D serum level increases, the age moves out of critical range and, above all, the degree of MS family kinship increases beyond the second. These findings are, in turn, useful for calculating the individual MS risk. In fact, R_MSf_ and R_MSm_ assume values of 0.099 and 0.097, respectively, corresponding to an age range of 20–45 years and normal vitamin D level of 20 nm/L. These values decrease quickly in the extreme ages of life. Finally, based on this evidence, we can now determine the health insurance premium in subjects at risk for MS. However, we must also consider biological aspects of these findings obtained through the proposed method. The latter, in fact, represent a suitable tool to describe, for the first time, the interaction between different biological variables and the consequent deterministic role in disease event occurrence, according to a mathematical function, with a known membership value.

In future perspectives, this study should be standardized in the extended population, finally providing the epidemiological basis of the personalized medicine in people at risk for MS.

## Figures and Tables

**Table 1:** The non-additive measure m on the index set N. Values *m*(S) represent the degree of disease risk corresponding to a medical condition with subset (S) indicating extremely abnormal risk factors.

	Female	Male
*m*(Ø)	0.000	0.000
*m*({1})	0.055	0.049
*m*({2})	0.071	0.065
*m*({3})	0.055	0.049
*m*({4})	0.095	0.098
*m*({1, 2})	0.081	0.074
*m*({1, 3})	0.071	0.065
*m*({1, 4})	0.095	0.098
*m*({2, 3})	0.076	0.065
*m*({2, 4})	0.110	0.108
*m*({3, 4})	0.095	0.098
*m*({1, 2, 3})	0.103	0.075
*m*({1, 2, 4})	0.115	0.113
*m*({1, 3, 4})	0.105	0.103
*m*({2, 3, 4})	0.110	0.108
*m*({1, 2, 3, 4})	0.121	0.117

**Table 2:** Demographic and clinical characteristics of the study population. Values for age at MS onset, vitamin D serum levels, and BMI are shown as mean ± standard deviation, with 95% confidence intervals in parentheses. * Kindship description: “*First degree*” means parent to child; “*Second degree*” means grandparent to grandchild or brother to sister; “*Third*, *fourth*, *fifth*, *sixth degrees*” include a nephew or niece to uncle or aunt, first cousins, children of first cousins or parent’s cousins, children of children’s cousins or children of parents’ cousins, respectively.

**N** (patients)	596
**Sex ratio** (female/male)	2.1/1.0
**Age at onset** (years)	32 ± 11.8 (20–45)
**Vitamin D serum levels** (nm/L)	28 ± 8.9 (10–30)
**BMI** (Kg/m^2^)	17 ± 8.6 (15–30)
**Patients with MS family kinship** (*n*) *	65
* First degree*	12
* Second degree*	26
* Third degree*	8
* Fourth degree*	8
* Fifth degree*	6
* Sixth degree*	5

**Table 3:** R_MSf_ and R_MSm_ for different female and male patients’ risk factors.

	Risk Factors				MS Risk Index	
	Age (x_1_)	Vitamin D (x_2_)	BMI (x_3_)	Kinship (x_4_)	R_MSf_	R_MSm_
**“p1”**	15	20	15	2	0.088	0.087
**“p2”**	30	20	15	2	0.099	0.097
**“p3”**	15	10	15	2	0.108	0.104
**“p4”**	30	10	15	2	0.110	0.106
**“p5”**	30	20	15	2	0.099	0.097
**“p6”**	30	20	30	2	0.106	0.102
**“p7”**	30	10	15	2	0.110	0.106
**“p8”**	30	10	30	2	0.117	0.109
**“p9”**	30	20	15	3	0.090	0.087
**“p10”**	30	20	15	1	0.107	0.107
**“p11”**	30	10	15	3	0.103	0.099
**“p12”**	30	10	15	1	0.117	0.114

“p1–p4”: R_MSf_ and R_MSm_ were calculated for each p1–p4 patient case, having fixed BMI = 15 kg/m^2^ and degree 2 of family kinship. In particular, p1–p2, p2–p3, and p3–p4 are the patient cases of increasing age (from ages 15 to 30), decreasing vitamin D serum level (from 20 to 10 nm/L), and the association of these two conditions, respectively. “p5–p8”: R_MSf_ and R_MSm_ were calculated for each p5–p6 patient case, having fixed age = 30 and degree 2 of family kinship. In particular, p5–p6, p6–p7 and p7–p8 are the patient cases of increasing BMI (from 15 to 30), decreasing vitamin D serum level (from 20 to 10 nm/L) and the association of these two conditions, respectively. “p9–p12”: R_MSf_ and R_MSm_ were calculated for each p9–p12 patient case, having fixed age = 30 and BMI = 15. In particular, p9–p10, p10–p11, and p11–p12 are the patient cases of increased family kinship (from 3 to 1 degree), decreasing vitamin D serum level (from 20 to 10 nm/L), and the association of these two conditions, respectively. For example, note that as regards a woman having a BMI of 15, a second degree of family kinship for MS and age ranging from 15 to 30 years, the major developmental contributor for disease results in the low vitamin D serum level. Furthermore, the major MS risk is conferred by the combination of increasing age with decreasing vitamin D serum level. In fact, R_MSf_ undergoes an increase of 13%, 23%, and 25% as well as R_MSm_ of 11%, 20%, and 22% for age going from 15 to 30 years, vitamin D serum level going from 20 to 10 nm/L, and for the association of these two conditions, respectively.

## Data Availability

Not applicable.
